# Severe Hiatal Hernia as a Cause of Failure to Thrive Discovered by Transthoracic Echocardiogram

**DOI:** 10.1155/2016/3821470

**Published:** 2016-11-08

**Authors:** Clint J. Moore, Devan A. Conley, Cristóbal S. Berry-Cabán, Ryan P. Flanagan

**Affiliations:** Womack Army Medical Center, Fort Bragg, NC 28310, USA

## Abstract

A newborn infant with failure to thrive presented for murmur evaluation on day of life three due to a harsh 3/6 murmur. During the evaluation, a retrocardiac fluid filled mass was seen by transthoracic echocardiogram. The infant was also found to have a ventricular septal defect and partial anomalous pulmonary venous return. Eventually, a large hiatal hernia was diagnosed on subsequent imaging. The infant ultimately underwent surgical repair of the hiatal hernia at a tertiary care facility. Hiatal hernias have been noted as incidental extracardiac findings in adults, but no previous literature has documented hiatal hernias as incidental findings in the pediatric population.

## 1. Introduction

Hiatal hernia (HH) in children is a well-recognized finding commonly diagnosed by both radiologists and gastroenterologists [1]. The prevalence of HH varies widely due to inconsistency in the definition; however, it is commonly defined as any herniation of elements in the abdominal cavity through the esophageal hiatus of the diaphragm [2, 3]. HHs are broadly divided into two classifications: sliding and paraesophageal hernias—with paraesophageal further divided into three classifications [2, 3]. HH as a congenital defect is largely associated with ill-defined feeding problems in infants and children. If left unrecognized, consequences may lead to malnutrition and growth suppression or failure to thrive (FTT) [4]. FTT among infants is directly related to growth failure, specifically with respect to the infant's ability to gain weight appropriately [4]. While many factors may contribute to FTT, the underlying cause remains as insufficient caloric intake [2–4]. Gastroesophageal reflux (GER) is a large contributing factor to infant malnutrition as children experience frequent vomiting from GER [4]. GER is common in patients with HH due to alterations of the anatomical relationship between the diaphragmatic esophageal hiatus and the stomach with cephalic displacement of the lower esophageal sphincter [5].

Although the diagnosis of HH is typically made by radiologists and gastroenterologists, findings of HH have also been recorded through incidental extracardiac findings during routine cardiac imaging procedures in adults [6]. These findings are often found through modalities such as cardiac magnetic resonance imaging, cardiac computed tomography, and myocardial perfusion imaging [1, 7]. This paper presents the first report of a patient who presented with FTT caused by HH, initially visualized by transthoracic echocardiogram (TTE).

## 2. Case Report

A healthy newborn infant female was referred to the pediatric cardiology for evaluation of a 3/6 harsh pan systolic murmur best heard at the lower left sternal border three days after birth during a routine early discharge exam. A TTE at that time revealed a 4 mm perimembranous ventricular septal defect (VSD) with left to right shunting, peak gradient of 18 mmHg, and a small anterior muscular VSD with left to right shunting. Additionally an anomalous vertical vein draining to the innominate vein was identified on the initial imaging, presumed to represent partial anomalous pulmonary drainage of the left upper lobe. Four other separate pulmonary veins were seen returning normally to the left atrium.

The patient was born at 40 weeks to a 26-year-old mother, G2P1, via low transverse caesarian section. The Apgar score was 9/9 and body weight 3340 g. An anatomy scan at 20 weeks showed no abnormalities. The patient's mother had three second trimester ultrasounds—all showing no abnormalities.

**Figure 1:**
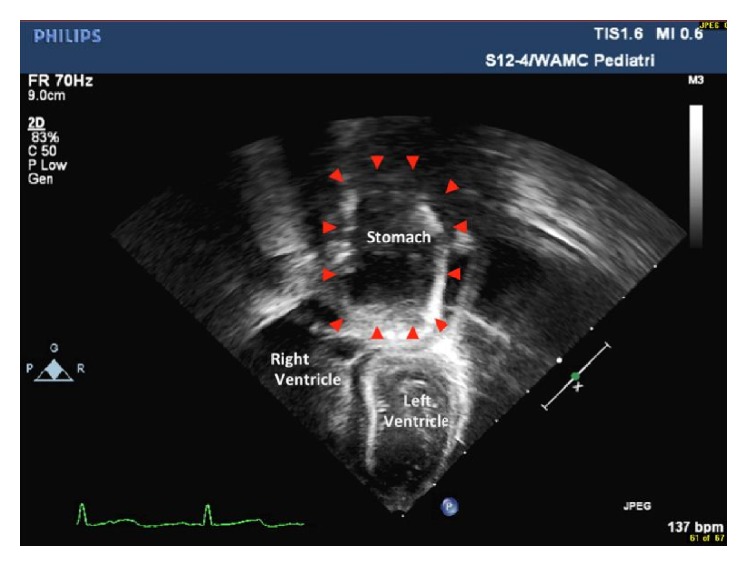
Modified apical 4-chamber view (posterior angulation) showing the stomach (arrowhead) above the left ventricle in the thorax outside of the pericardium.

**Figure 2:**
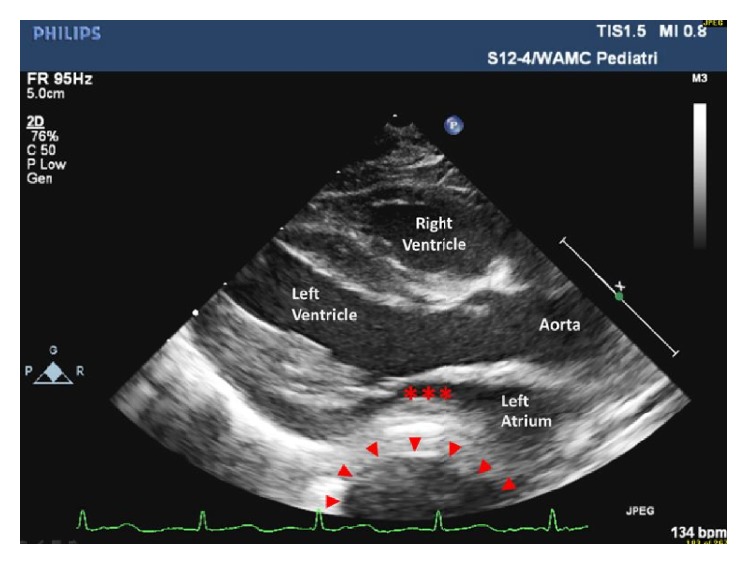
Parasternal long axis showing anterior deviation of the left atrium with anatomical impingement of the mitral valve annulus (*∗*) by the stomach (arrowhead).

At the patient's two-week checkup she was found to have lost weight. The parents reported trouble feeding with poor latch and agitation with vomiting after eating and occasional panting. The patient's weight was down 5% from birth. Pediatric cardiology reevaluated the patient due to poor feeding, failure to gain weight, persistent murmur, and previously suspected anomalous pulmonary venous return. A repeat TTE showed restrictive perimembranous VSD (peak gradient > 60 mmHg) and an anomalous vein draining to the innominate vein suggestive of partial anomalous pulmonary venous return. Additionally, there was an interval change with a prominent fluid filled structure posterior to the left atrium on echocardiogram (Figures 1 and 2). The structure was approximately one-half the size of the left atrium situated just posterior to the left atrioventricular groove. The intrathoracic fluid filled structure appeared to be extrapericardial. Her symptoms were not felt to be secondary to the VSD or PAPVR because the VSD was small and pressure restrictive and the PAPVR was only one single vein with at least four other veins returning to the left atrium.

**Figure 3:**
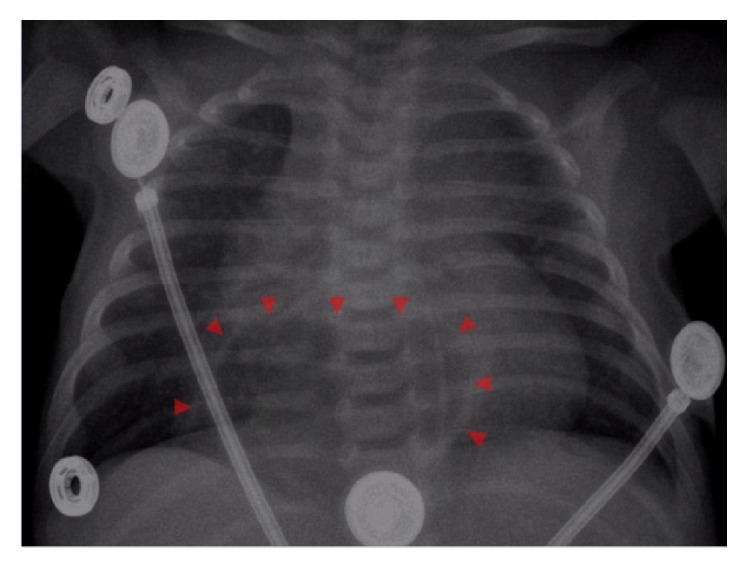
AP chest X-ray showing a radiolucency over the cardiac silhouette just above the diaphragm (arrowhead).

**Figure 4:**
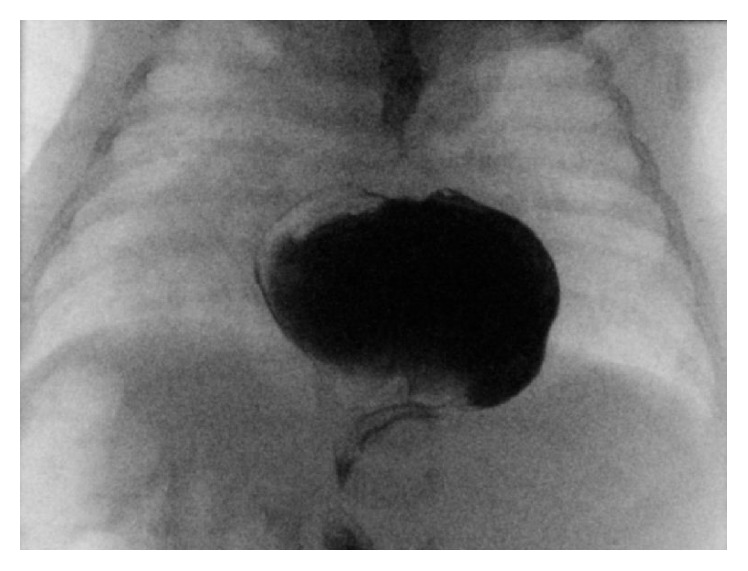
Definitive imaging (upper GI series) showing the majority of the stomach above the diaphragm.

Due to the new unclear fluid filled thoracic structure, previous finding of partial anomalous pulmonary venous return, and presenting signs/symptoms she was transferred to a tertiary care center for more definitive thoracic imaging. At the tertiary center, a chest radiograph revealed a radiolucency over the heart border (Figure 3) prompting CT angiogram that was suspicious for a large hiatal hernia. She subsequently underwent an upper gastrointestinal series that delineated the hiatal hernia as a large sliding hiatal hernia with the entire stomach above the diaphragm (Figure 4). The hiatal hernia resulted in delayed gastric emptying and gastroesophageal reflux disease. This was thought to be the cause of her failure to thrive and tachypnea. A nasoduodenal tube was placed to optimize weight gain before surgery and she was given 24 kcal fortified expressed breast milk. Patient was started on omeprazole and discharged after 24 hours of no emesis and good somatic weight gain (24 g/day). Neither the VSD, nor her anomalous pulmonary venous return was believed to be hemodynamically significant.

The patient ultimately underwent surgical repair of her hiatal hernia with Nissen fundoplication and gastric tube placement. At four-month follow-up her parents reported that she was doing well taking all of her feeds by mouth. She was gaining weight along the World Health Organization Growth Standards with documented catch up weight gain. Parents reported no further issues with breathing or feeding. She has had multiple subsequent follow-up evaluations in pediatric cardiology. Although the VSD and PAPVR has persisted neither appear to be hemodynamically significant. The VSD peak instantaneous pressure gradient has increased (>90 mmHg) and no evidence of chamber enlargement has been noted on her echocardiograms.

## 3. Discussion

Incidental extracardiac findings by transthoracic echocardiogram have been described in adults [6, 7]. The most common extracardiac findings in those studies were hepatic findings, pleural effusions, and anomalies of the descending aorta (dilation, atheroma, ulcer, or thrombus). Although hiatal hernia was reported as an extracardiac finding in one of these case series, this is the first description of a hiatal hernia identified by transthoracic imaging in an infant. This patient's presentation is consistent with a large hiatal hernia but the method in which it was discovered has not been previously reported.

Multiple works have shown cross-sectional imaging studies, such as CT, myocardial perfusion imaging, and Cardiac MRI, to have a higher prevalence of incidental findings [6, 7]. Despite a lower prevalence on transthoracic echocardiograms a retrospective review of transthoracic echocardiograms in adults found that 7.5% of all studies analyzed had an incidental extracardiac finding [7]. That same study showed that more than half of these findings altered the management of the patient [7].

Despite the multiple reports in adults there is a paucity of data in pediatric cardiology with respect to incidental findings on transthoracic echocardiograms. Future works could attempt to describe the prevalence in pediatric cardiology. Despite the recommendation that all incidental findings during a pediatric echocardiogram should be noted in the report [8], previous reports have shown that extracardiac findings during transthoracic echocardiograms are reported as little as 22% of the time [7]. This case highlights the importance of constant vigilance during any imaging study to ensure that clinically significant incidental findings are not overlooked.
